# Effect of 10.6-μm CO_2_ laser moxibustion on the fastest 15-m walking time in patients with knee osteoarthritis: a double-blind, sham-controlled, multi-site randomized trial

**DOI:** 10.1186/s13018-023-04380-6

**Published:** 2023-11-22

**Authors:** Lusheng Chen, Ling Zhao, Ke Cheng, Lin Lin, Fan Wu, Xueyong Shen

**Affiliations:** 1 Shanghai Key Laboratory of Acupuncture Mechanism and Acupoint Function, Fudan University, Shanghai, 201433 China; 2 School of Acupuncture-Moxibustion and Tuina, Shanghai University of Traditional Chinese Medicine, No. 1200 of Cailun Road, Pudong New District, Shanghai, 201203 China; 3 Shanghai Research Center of Acupuncture and Meridian, 421 Niudun Road, Shanghai, 201203 China

**Keywords:** Acupuncture therapy, CO_2_ laser moxibustion, Knee joint, Osteoarthritis, Walking time

## Abstract

**Background:**

In this study, we investigated the impact of 10.6-μm CO_2_ laser moxibustion (LM) on the fastest 15-m walking time in individuals suffering from knee osteoarthritis (KOA).

**Methods:**

A total of 392 individuals diagnosed with KOA and meeting the specified eligibility criteria were assigned randomly into two groups: the LM treatment group and the sham LM control group (ratio 1:1). Both groups received either LM therapy or simulated LM therapy to address the affected area of the knee joint. This treatment was administered three times a week for a duration of 4 weeks.

**Results:**

In the LM group, the fastest 15-m walking times at both Week 4 and Week 12 were significantly reduced compared to the times before treatment (all *P* < 0.05). However, in the sham LM group, there were no notable differences in the fastest 15-m walking times after treatment (all *P* > 0.05). Moreover, the LM group exhibited shorter 15-m walking times than the sham LM group at both Week 4 and Week 12 (all *P* < 0.05).

**Conclusion:**

The use of CO_2_ LM can lead to a substantial enhancement in the fastest 15-m walking time of individuals suffering from KOA, and its therapeutic impact can last for a minimum of 8 weeks post-treatment. The fastest 15-m walking time serves as an indicator of alterations in the walking capacity of patients with KOA.

## Introduction

Knee osteoarthritis (KOA) is a common source of impairment in the elderly [1]. KOA affects 12.1% of people aged 60 and above in the USA [2–4]. As people get older, their chances of having KOA increase [5]. KOA affects 30% of the population in China, with the condition being more prevalent among females than males [6–8]. Degenerative alterations in the knee joint cause clinical symptoms such as joint enlargement, edema, softness of the patellofemoral joint, and other problems. Furthermore, individuals frequently feel decreased joint mobility, tingling sensations within the joint, pain while bearing weight, and difficulty when climbing stairs [9]. These symptoms, especially pain and functional restrictions, have a substantial impact on the quality of life of individuals and can make walking challenging. Assessing the walking capability of patients with KOA hinges significantly on their fastest walking time. Typically, the standard distance for measuring fastest walking time in KOA cases ranges between 40 and 50 m [10–13]. However, subjecting patients to long-distance fast walking can impose greater strain on those with KOA, potentially causing additional motor damage, which is counterproductive for managing the condition. Moreover, in most hospitals, there is a scarcity of accessible walkways nearly 50 m in length for conducting such tests.

White et al. examined gait speed over a distance of 20 m in patients with KOA aged 45–79. Their findings revealed a significant reduction in gait speed at this shorter distance among patients experiencing pain symptoms [14]. Similarly, Gilbert et al. conducted a comparison between two gait speeds, 20 m and 400 m, in patients with KOA. Their results indicated that gait tests over a shorter 20-m distance were consistent with the differences in symptoms experienced by patients [15]. Using a shorter distance for walking tests can help mitigate risks and enhance patient compliance. Low-level laser therapy has been widely used to treat musculoskeletal pain including pain in knee OA. We have developed a laser moxibustion (LM) device of 10.6 μm wavelength, which has the thermal nature of moxibustion without smoke and smell. The LM device was patented in 2010 (China Invention Patent ZL200910056991.4) and licensed by Shanghai Municipal Food and Drug Administration, China (20162210783). This trial aimed to determine whether 10.6-μm CO2 laser treatment could improve 15-m fastest walking time in individuals with KOA, and the findings are presented in the following.

## Materials and methods

### Ethical approval and protocol registration

This research involved a double-blind, sham-controlled, multi-site randomized trial. The details of this trial can be accessed at this URL: https://doi.org/10.1186/ISRCTN15030019. The ISRCTN trial registration identifier is 15030019. The Institutional Review Board (IRB) at each of the six hospitals where the research was conducted gave their clearance. The study was conducted in outpatient clinics in these hospitals.

### Sample size and recruitment

Previous studies revealed that laser moxibustion showed a significant improvement rate of 14.1% in fast walking time, while the sham laser moxibustion group only exhibited a rate of 4.8%. There was a significant difference between the two groups (*P* < 0.01) [13]. It is important to note that these findings were obtained through collaboration with a knee osteoarthritis research specialist, as well as a statistician, ensuring both clinical and statistical expertise.

To determine the appropriate sample size, a power analysis was conducted using PASS20.0.1 software (Power Analysis and Sample Size, PASS20 NCSS), considering a significance level (*α*) of 0.05 and a power (1 − *β*) of 80%. The analysis indicated that each group required a minimum of 151 participants. However, considering the possibility of dropouts and potential insufficiency in case numbers, it may be necessary to recruit additional cases in order to maintain an adequate sample size.

**Fig. 1:**
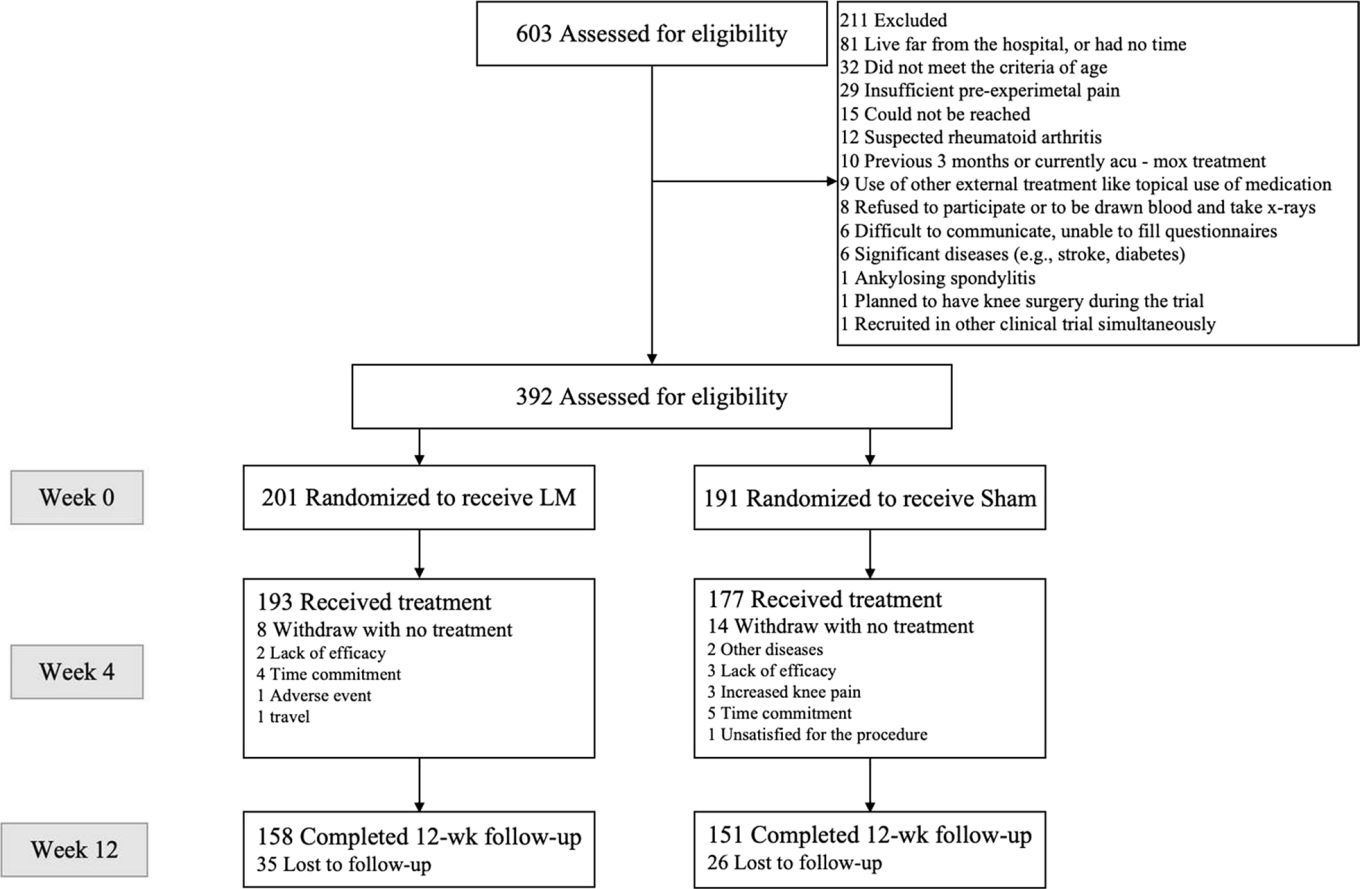
Participant flowchart

Between January 2015 and November 2017, a total of 603 individuals were assessed for participation, primarily recruited through print ads in local newspapers and posters distributed in nearby communities (as illustrated in Fig. 1). Out of this initial pool, 211 individuals were excluded from the study. Among the remaining participants, 22 individuals dropped out of the treatment specified in the protocol [16], citing various non-therapeutic reasons, while 61 individuals did not complete the follow-up process. Consequently, a group of 309 participants successfully completed the entire 4-week treatment and follow-up period. This cohort included 158 participants in the LM group and 151 participants in the sham LM group. All participants provided written informed consent, and we ensured their full comprehension of the study. To guarantee data safety and maintain trial quality as well as patient safety, we established an international Data and Safety Monitoring Board (DSMB).

### Eligibility criteria, randomization, and blinding

#### Inclusion criteria

Prior to enrollment, a specialist doctor conducted an assessment of knee joint function in the patients. Anteroposterior and lateral X-ray images were obtained for the affected joint, and inclusion criteria were applied to select the subjects for the study.

1. Age 50 to 75 years.
2. According to the American College of Rheumatology criteria for the diagnosis of knee osteoarthritis [17–21].
3. Radiological confirmation of knee osteoarthritis (Kellgren–Lawrence grade ≥ 1) [22].
4. Moderate or worse knee pain most time during the past month; subjects had a vas baseline score of 40 or greater for arthritic pain.
5. Agree to be randomized and understand and be willing to sign the informed consent.

#### Exclusion criteria

Individuals with knee-related conditions such as rheumatoid arthritis, fibromyalgia syndrome, chronic fatigue syndrome, and ankylosing spondylitis were not included in the study. Additionally, individuals who met the following criteria were also excluded.

1. Those who had taken steroid medication or undergone acupuncture/moxibustion treatment in the last 3 months.
2. Those who had received intra-articular hyaluronate injections within the past 6 months.
3. Those who had undergone arthrocentesis or arthroscopy in the last year.
4. Those with a history of knee/hip replacement surgery or plans for such surgery during the trial.
5. Those using other external treatments, such as topical medications.
6. Individuals with serious medical conditions, including cardiac diseases, pulmonary diseases, kidney diseases, liver diseases, malignant tumors, systemic infections, contagious diseases, or psychopathy.
7. Those who had used the study treatment in the previous 30 days.
8. Individuals who had previously participated in other laser therapy trials.
9. Participants recruited simultaneously for another clinical trial.
10. Those unable to complete measurement questionnaires.

### Randomization and blinding

A total of 392 eligible participants were divided into two groups: the LM group and the sham LM group. The allocation of patients to these groups was determined through a computer-generated randomization sequence. The subjects were assigned numbers based on the order in which they completed the forms. A statistician generated a random sequence of numbers using the seed number 2605 in the STATA/SE11 software. The random sequence comprised random numbers, which were then sorted either in ascending or descending order based on their magnitude. The original codes associated with the sorted order were utilized as the basis for grouping. In order to maintain the confidentiality of this randomization process, the central randomization system generated letter codes (designating either LM equipment or sham LM equipment), which were then transmitted to the field coordinator. Once the equipment operator received the equipment code from the current field coordinator, they used the equipment bearing that code to treat the patient. It is important to note that both types of equipment emitted the same red light during operation, making it impossible for the operator to discern which group the equipment belonged to. The entire procedure was closely monitored by the coordinator to ensure that the study adhered to the established protocol. Trained operators administered treatment to both groups in a dedicated room to prevent any communication among patients. Consequently, no one involved in the study, including patients, equipment operators, outcome assessors, study coordinators, and statisticians, was aware of the treatment assignments.

### Intervention

#### CO_2_ laser therapeutic instrument

In this clinical study, the SX10-C1 CO_2_ laser moxibustion (LM) therapeutic apparatus, a collaborative development between Shanghai Wanqi Photoelectric Technology Co., Ltd., and Shanghai University of Traditional Chinese Medicine was employed. The laser generator within this apparatus has the capability to emit an infrared laser with adjustable output power ranging from 160 to 180 milliwatts and a wavelength of 10.6 µm. Each treatment session delivers an energy output in the range of 61.2–68.8 J/cm^2^. The placebo therapy equipment shares an identical appearance with the CO_2_ LM therapy equipment but does not produce any laser output during sham treatments. Furthermore, it is worth noting that the LM device obtained a patent in 2010 (China Invention Patent ZL200910056991.4) and received approval from the Shanghai Municipal Food and Drug Administration, China (20162210783).

#### Duration and method of treatment

Participants were positioned in a supine posture with both knees exposed and padded below the popliteal fossa using a small pillow to maintain a slightly bent and relaxed position. Two specific acupoints were chosen, namely the Dubi point (ST35, situated on the outer side of the patella and in the recess of the patellar ligament) and the Ashi point (a tender point) in the affected knee [23]. Radiation therapy was administered for a duration of 20 min, three times per week for a total of four weeks (comprising a total of 12 sessions). If any treatment session was missed, it was rescheduled within the same week. The treatment course was not extended, and a maximum of two missed sessions were allowed; otherwise, the patient was considered withdrawn from the trial.

#### Outcome

The fasting 15-m walking time was recorded prior to the intervention, during Week 4, and at Week 12.

#### Statistical methods

The statistical analysis of the data involved was conducted using SPSS version 22.0 software. To assess whether the data followed a normal distribution, the Wilcoxon test was employed. If the data exhibited a normal distribution, it was presented as the mean ± standard deviation. Alternatively, for data that did not adhere to a normal distribution, it was presented as median values (Q1, Q3). Gender and knee condition comparisons between groups were conducted using the Chi-squared test, while general measurement data comparisons utilized the *t*-test. To examine the intra-group effect of the fastest 15-m walking time before and after treatment in both groups, repeated measures analysis of variance was applied. Finally, multiple analysis of variance was used for assessing the inter-group effect.

## Results

**Fig. 2:**
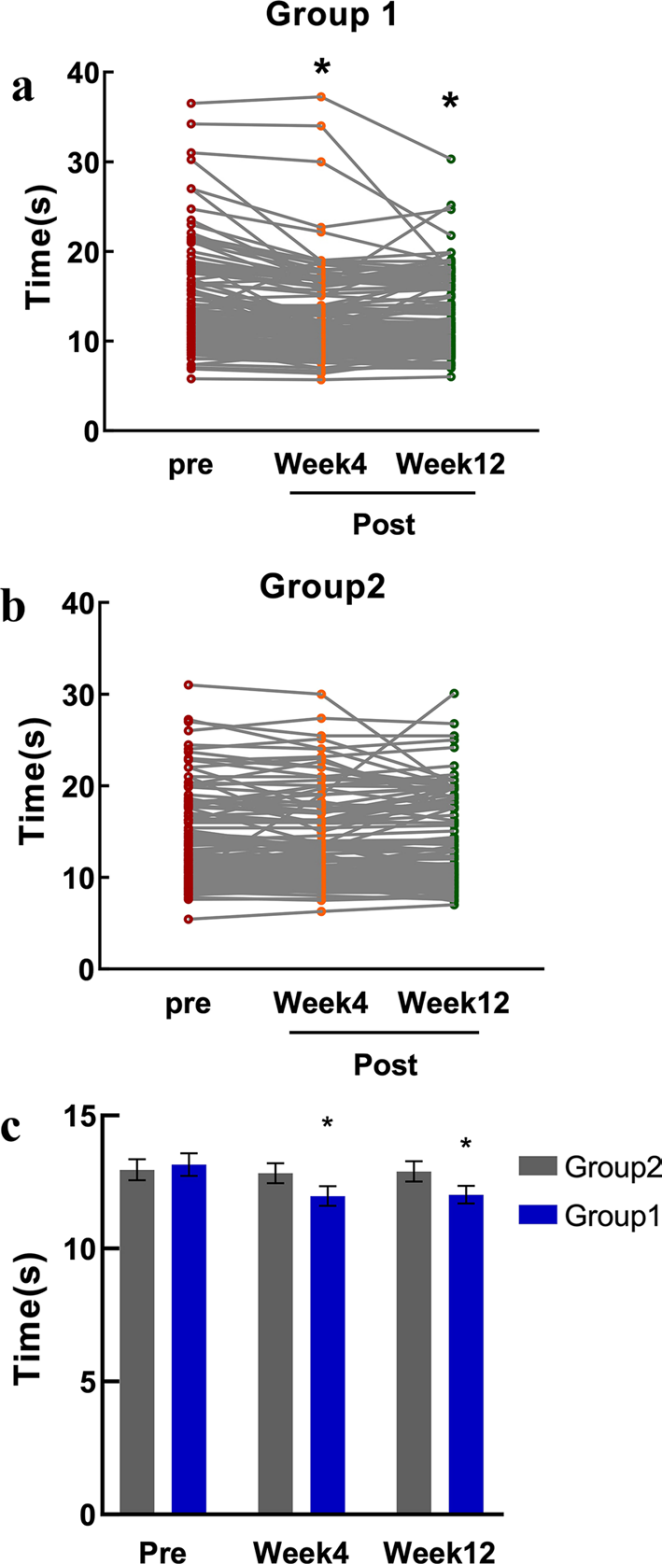
Comparison of 15-m fast-paced walk time. **a** ^*^LM group compared with before treatment, at the 4th and 12th weeks, *P* < 0.05. **b** There was no significant difference between the 4th and 12th weeks before treatment in the sham LM group. **c** ^*^The comparison between the two groups at the 4th and 12th week, *P* < 0.05

There were no notable differences in terms of gender, age, disease duration, body mass index (BMI), and affected joints observed between the two groups (*P* > 0.05), indicating that the two groups were similar (Table 1). As indicated in Table 2 and Fig. 2, there was no notable difference in the initial 15-m walking time prior to treatment between the two groups (*P* > 0.05). However, the 15-m walking time significantly decreased at various post-treatment time points in the LM group compared to their pre-treatment values (all *P* < 0.05). Conversely, in the sham LM group, there was no significant variation in the 15-m walking time measured at each post-treatment time point when compared to their pre-treatment values (all *P* > 0.05). Notably, during the follow-up at Week 4 and Week 12, the 15-m walking time in the LM group was significantly shorter than that in the sham LM group, and these differences held statistical significance (all *P* < *0.05*).

**Table 1:** Demographic data comparison

Group	LM group (n = 158)	Sham LM group (n = 151)
*Age*	64.74 ± 6.38	63.93 ± 5.86
Female (%)	123 (77.85)	109 (72.19)
*Affected joint (%)*		
One joint	62 (39.24)	72 (47.68)
Two joints	96 (60.76)	79 (52.32)
*Disease duration (%)*		
1 years	30 (18.99)	20 (13.25)
1–5 years	72 (45.57)	69 (45.70)
5–10 years	20 (12.66)	22 (14.57)
10 years	36 (22.78)	40 (26.49)
*BMI*	24.98 ± 3.68	24.64 ± 9.15

*LM* laser moxibustion, *BMI* body mass index

**Table 2:** Comparison of 15-m fast-paced walking time

Weeks	*N*	LM group		*N*	Sham LM group		*Z* value	*P* value
		(Q1, Q3) Median	95% CI		(Q1, Q3) Median	95% CI		
0	158	11.2900 (9.7300, 15.5975)	12.3217, 13.9909	151	11.0000 (9.5300, 16.0000)	12.1879, 13.7357	− 0.322	0.747
4	158	10.4650 (8.9200, 13.6550)	11.2432, 12.6982	151	10.8000 (9.5500, 15.4000)	12.0794, 13.5805	2.228	0.026
12	158	10.3800 (8.9075, 15.0400)	11.3666, 12.6725	151	10.6800 (9.5800, 16.1800)	12.1435, 13.6476	2.205	0.027

*LM* laser moxibustion

## Discussion

KOA, referred to as “Knee Arthralgia” in traditional Chinese medicine (TCM), falls under the category of “Bi syndrome” in TCM [24]. The development of KOA is influenced by factors such as age, gender, trauma, and others. This condition is more prevalent among older individuals and is characterized by degenerative changes and increased bone growth in the knee joint. Degenerative lesions, on the other hand, are typically associated with irregularities in bone metabolism. In TCM, a common treatment for KOA is moxibustion, which has demonstrated significant efficacy in managing this condition [25]. Moxibustion exerts its therapeutic effects primarily through the application of heat and light. When moxibustion is ignited, it emits infrared radiation within a specific wavelength range, stimulating acupuncture points, promoting anti-inflammatory responses, and providing relief from pain. Our initial research revealed that LM can provide therapeutic benefits in the rat model of KOA induced by monosodium iodoacetate, resulting in improved mechanical pain tolerance and load-bearing capacity in rats (*P* < 0.001). Simultaneously, it reduces the levels of MMP-13 in the cartilage, as well as TNF-α, IL-1β, and IL-6 in the synovium (*P* < 0.05), exhibiting analgesic, anti-inflammatory, and cartilage-protective effects [26]. Furthermore, LM alleviates KOA pain by suppressing neuroinflammation mediated by microglia activation in the rat spinal cord [27]. These findings suggest that LM is a promising treatment for KOA.

Building on this foundation, we conducted a randomized, double-blind controlled trial involving LM. After a four-week treatment period, we observed significant improvements in pain symptoms in individuals with KOA, as indicated by the Western Ontario and McMaster Universities Osteoarthritis (WOMAC) score (*P* < 0.01). Additionally, the increase in serum Cartilage Oligomeric Matrix Protein (COMP) in these individuals benefits the protection and repair of articular cartilage [25]. The relief of pain and cartilage repair are closely associated with knee joint function.

Furthermore, our research team conducted separate randomized, double-blind, and controlled clinical trials for moxibustion and LM. Both interventions led to a substantial improvement in the fastest walking time (46 m or 50 yards) for patients with KOA before and after treatment. Consequently, we posit that LM enhances the walking ability of patients with KOA by alleviating pain and enhancing cartilage health. Our primary research objective is to investigate whether LM can influence the shortest walking time. The current method for detecting the fastest walking time involves having patients walk 50 yards as fast as possible. However, this can lead to knee joint injuries, pain, and falls in patients, causing them to be reluctant to participate in the test and dropping out. In the early stages of our study, we decided to make the test safer and more reflective of the walking abilities of patients with KOA. As a result, we conducted the fastest walking time test over a shorter distance of 15 m in this study. This approach also revealed a correlation between the fastest walking time over a short distance and the pain and other symptoms experienced by patients with KOA, as indicated by previous studies [16, 28, 29].

The primary factors contributing to reduced walking ability in these patients are pain, stiffness, and dysfunction in the knee joint. Therefore, the key to their treatment lies in reducing inflammatory pain, alleviating stiffness, and enhancing knee function. Our study demonstrated that in the LM group, the fastest 15-m walking time was significantly shorter at Week 4 and Week 12 compared to before treatment. In contrast, there were no significant differences in the fastest walking time before and after treatment or between Week 4 and Week 12 in the sham LM group. When comparing the two groups, it is evident that the LM group showed greater improvement in the fastest walking time compared to the sham LM group. This indicates that LM treatment effectively enhances the fastest walking time over 15 m in patients.

On the other hand, sham LM did not result in any improvement in the fastest walking time. There were no significant differences observed before and after the end of treatment or at Week 4 and Week 12 in this group. These findings suggest that LM treatment has a lasting effect on KOA treatment, with improved walking ability persisting for at least 8 weeks after treatment completion. Future research could explore the specific duration of this efficacy through longer follow-up periods.

Overall, the study results highlight the significance of the 15-m walking distance as a means to assess the walking abilities of patients with KOA. The fastest 15-m walking test not only enhances the efficiency of healthcare providers but also encourages greater patient compliance, reduces risks, and supports its broader adoption and clinical application. This research, however, is subject to several limitations. Beginning with the fact that the research was conducted in six separate sites with differing numbers of recruited participants in each, selection bias and conditional bias could be introduced. Furthermore, in TCM, acupoint selection is based on the concepts of syndrome classification, whereas in this therapy, we only used two fixed acupoints. Furthermore, although patients were instructed to keep a drug use diary, we cannot guarantee that this instruction was followed to the letter. Although we believe randomization can solve this problem by guaranteeing that both groups have an equal number of participants, our requirements might not be followed exactly. As a result, we plan to adopt tougher techniques for regulating fundamental drug usage of patients in future studies and to more closely link our methodologies with clinical practice.

## Conclusion

The ability of patients with KOA to walk 15 m quickly can be significantly improved with the use of CO_2_ LM. Additionally, its therapeutic benefits can sustain for at least 8 weeks after the treatment. The fastest 15-m walking time serves as an indicator of the improvements in the mobility of patients with KOA.

## Data Availability

The datasets used and/or analyzed during the current study are available from the corresponding author on reasonable request.
